# RNA Interference of the Genes Associated with the Invasion of Brain Tumor Cells

**DOI:** 10.32607/actanaturae.27657

**Published:** 2025

**Authors:** M. A. Dymova, O. A. Kartina, D. V. Drokov, E. V. Kuligina, V. A. Richter

**Affiliations:** Institute of Chemical Biology and Fundamental Medicine, Siberian Branch of the Russian Academy of Sciences, Novosibirsk, 630090 Russia

**Keywords:** glioma, invasion, RNA interference, small interfering RNA

## Abstract

High-grade gliomas are among the most aggressive malignant pathologies of the brain. The high invasive potential of tumor cells causes relapses of the disease even after radical resection of the tumor. The signatures of the genes associated with the invasion of glioma cells have now been identified. The expression products of these genes are involved in various signaling pathways, such as cellular protein catabolism, the p53 signaling pathway, transcription dysregulation, and the JAK-STAT signaling pathway. Therefore, they can indirectly modulate the invasive potential of tumor cells. Using RNA interference technology, it is possible to change the expression level of the detected genes and reduce the invasive and proliferative potentials of cancer cells. This review focuses on the use of this technology to influence various links in signaling pathways and, accordingly, the cellular processes associated with the invasion of glioblastoma cells. Furthermore, the review discusses the problems associated with delivering interfering RNAs into cells and ways to solve them.

## INTRODUCTION

Glioblastoma (grade IV glioma) is an aggressive malignant pathology of the brain accounting for 49% of primary malignant tumors of the central nervous system [1]. The incidence of this tumor is approximately 10 cases per 100,000 people. The median survival of glioblastoma patients undergoing standard treatment is ~ 14 months; the five-year survival rate is as low as 7.2% [2]. There are several factors contributing to the low survival rate of patients with this cancer: (1) the infiltrative tumor growth pattern complicating its complete resection; (2) the high degree of genetic intratumor and intertumor heterogeneity, which hinders targeted therapy; (3) the blood–brain barrier (BBB) impeding drug delivery to tumor tissue; (4) the immunosuppressive tumor microenvironment inhibiting antitumor immunity; and (5) the lack of reliable methods for early disease diagnosis. Today, the standard glioblastoma treatment protocol comprises maximal safe resection of the tumor, temozolomide (TMZ) chemotherapy, and radiation therapy (the so-called Stupp protocol) [2]. Other chemotherapeutic agents are used along with TMZ: vincristine, lomustine, procarbazine [1], methotrexate [3], Gliadel [4], and paclitaxel [5, 6]. The extent of the surgical resection positively correlates with patient survival; however, the infiltrative tumor growth pattern, the blurred boundaries between the tumor and healthy tissue, and the consequential risk of damaging the healthy brain areas during surgery complicate complete tumor resection [2]. Temozolomide therapy also involves several problems, such as the development of drug resistance by the tumor cells, adverse events associated with myelosuppression, the short half-life of TMZ, and the low effectiveness in crossing the BBB (~ 20%), leading to the need for higher therapeutic doses and, consequently, more severe adverse events [7]. Therefore, searching for novel effective glioblastoma treatments remains one of the most pressing challenges facing practical oncology.

**Invasion as one of the hallmarks of glioblastoma**

A key hallmark of glioblastoma is the active invasion of tumor cells occurring along the existing structures, primarily along blood and lymphatic vessels and the walls of cerebral ventricles, or via direct penetration through the dura mater and bone. The ability of tumor cells to undergo reversible epithelial–mesenchymal transition (EMT) allows a remodeling of their cytoskeleton and amoeboid movement among other cells, thus altering the structure of the extracellular matrix [8, 9]. Metastatic cancer stem cells (mCSCs) stand out among the pool of glioma cells [10]. The epigenetic plasticity of mCSCs enables them to switch between the stationary, slow-proliferative (dormant) state and the migratory mesenchymal-like state. That is how the invasion of tumor cells into the adjacent niches and the formation of metastases, where mCSCs express mesenchymal subtype markers such as CD44 and YK-40, takes place.

Tumor cells are capable of releasing a glutamate neurotransmitter into the extracellular space, thus inducing excitotoxic death of surrounding neurons and making room for amoeboid movement. Microglial and tumor cells also secrete various enzymes (urokinase plasminogen activator, cathepsin B, as well as MMP and ADAM proteases), thereby degrading proteoglycans and hyaluronic acid in the extracellular matrix along blood vessels, making it possible for cells to enter the bloodstream [11]. The formation of dense cellular structures known as pseudopalisades, primarily composed of microglial cells and macrophages, is pathognomonic for glioblastoma [12]. Some tumor cells have lamellipodia; electrical synapses in them ensure intercellular communication and coordination [13].

**Genes associated with glioblastoma invasion processes**

**Table 1:** The genes associated with an invasion of glioma cells

No.	Signaling pathways and cellular processes	Gene	Reference
1	Regulation of cellular protein catabolism	CLU, HSP90AB3P, MDM2, OS9, SDCBP, TRIB2	[14, 15, 16, 17, 18, 19, 20]
2	The p53 signaling pathway	CASP3, CCND2, CDK4, IGFBP3, MDM2	[14, 17, 21, 22, 23, 24]
3	Regulation of transcription in cancer cells	CCND2, IGFBP3, MDM2, PLAT, ZEB1	[14, 17, 22, 24, 25, 26]
4	The JAK-STAT signaling pathway	CCND2, FHL1	[14, 22, 26]

Transcriptome analysis and single-cell DNA sequencing of glioma have helped identify the gene signatures (*Table 1*) associated with cancer cell invasion (metastasis- associated genes, MAGs) [14]. The products of these genes are involved in the p53 and JAK-STAT signaling pathways, as well as in cellular processes such as the catabolism of cellular proteins and regulation of transcription, differentiation, and the proliferation of cells. Suppression of the expression of these genes may contribute to a reduction of both the invasive and the proliferative potential of glioma cells.

Furthermore, Cox regression analysis revealed another three genes (*GNS*, *LBH*, and *SCARA3*) whose expression correlates with the survival time of patients diagnosed with IDH-wildtype glioma [14, 27, 28]. The *GNS *gene encodes glucosamine (N-acetyl)-6- sulfatase, which is involved in the catabolism of heparin, heparan sulfate, and keratan sulfate. The *LBH* gene is highly expressed in gliomas. Under hypoxic conditions, its expression is directly regulated by the transcription factor HIF-1 and promotes tumor angiogenesis. The *SCARA3 *gene encodes the scavenger receptor class A member 3 that reduces the level of reactive oxygen species, thereby protecting cells against oxidative stress.

Since tumor cell invasion is considered to be a key prognostic factor of the disease, it is crucial to identify the transcription factors, signaling pathways, and key master regulators of this process both for understanding the molecular mechanisms of oncogenesis and for further developing targeted therapeutics for glioma treatment.

**RNA interference as a therapeutic approach**

RNA interference, a natural evolutionarily conserved cellular defense mechanism against foreign gene invasion, which is commonly found in organisms across various taxa, is one of the gene expression regulation methods [29]. RNA interference is the post-transcriptional suppression of gene expression through degradation of their mRNA triggered by small non-coding RNAs complementary to the mRNA sequence. These non-coding RNAs include double-stranded small interfering RNAs (siRNAs) and single-stranded short hairpin RNAs (shRNAs). Eukaryotic cells contain the DICER enzyme that hydrolyzes long endogenous and exogenous double-stranded RNAs into shorter fragments and cleaves the shRNA loop, yielding short siRNAs. siRNA binding to the target mRNA results in the formation of the RNA-induced silencing complex (RISC), which is involved in enzymatic mRNA degradation and suppresses translation [30, 31]. Unlike synthetic siRNAs, which are delivered into cells as short double-stranded RNAs, plasmid DNA or viral vectors are typically utilized in the case of shRNAs. After they have been delivered into the cell, shRNA is transcribed in the cytoplasm and converted to functional siRNA by the DICER enzyme.

**Table 2:** FDA-approved siRNA-based therapeutics

Therapeutic	Indications for use	Target	Delivery system	Year of FDA approval
Patisiran	Familial amyloid polyneuropathy	Hepatic transthyretin	Liposomes	2018
Givosiran	Acute hepatic porphyria	Aminolevulinic acid synthase 1	NAcGal	2019
Lumasiran	Primary hyperoxaluria type 1	Hepatic glyoxylate oxidase	NAcGal	2020
Inclisiran	Hypercholesterolemia	Subtilisin/kexin type 9	NAcGal	2021
Vutrisiran	Hereditary transthyretin amyloidosis with polyneuropathy	Transthyretin	NAcGal	2022
Nedosiran	Primary hyperoxaluria	Hepatic lactate dehydrogenase	NAcGal	2023

RNA interference is a gene therapy method for various diseases. Six siRNA-based therapeutics have been approved for clinical application (*Table 2*). In 2018, the U.S. Food and Drug Administration (FDA) and the European Medicines Agency (EMA) approved patisiran as the first siRNA-based therapeutic for treating polyneuropathy caused by hereditary transthyretin amyloidosis in adult patients. Another six siRNA-based therapeutics have successfully undergone clinical trials. Fitusiran (NCT05662319), teprasiran (NCT03510897), and tivanisiran (NCT05310422) [32] are currently undergoing phase III clinical trials.

**Problems related to the application of siRNA in targeted therapy**

Despite the high potential of RNA interference-based therapy, the availability of therapeutics approved for clinical application and several promising clinical trials, the RNA interference technology continues to exhibit a number of fundamental limitations. Significant challenges in the clinical application of interfering RNAs include nuclease degradation of unbound nucleic acids in bodily fluids, rapid renal clearance, interaction with extracellular proteins, and poor cellular internalization efficiency [33]. Along with the biopharmaceutical properties, the physicochemical characteristics of these molecules (their hydrophilicity, negative charge, and instability) also substantially hinder siRNA delivery into cells and reduce their biological activity [34]. Nucleic acids *per se *are neither tissue- nor cell-specific and poorly penetrate across various biological barriers, thus impeding the development of orally, intranasally, or transdermally administered drugs based on them [33]. Furthermore, off-target effects of RNA interference have been observed [35]. Thus, administration of shRNA targeting* HCN1 *mRNA into different brain regions of mice induced cytotoxicity mediated by them, including hippocampal cell degeneration even when delivering the control shRNA targeting luciferase mRNA (whose gene is absent from the mouse genome) [36]. These off-target effects of RNA interference may arise from both the binding of siRNA seed regions to the 3’-untranslated regions of non-target mRNAs, leading to their cleavage by the DICER complex, and the fact that the delivery of additional exogeneous RNA into the cell triggers competition with endogenous RNAs at all interference stages (e.g., for binding to DICER and RISC complexes in the cytoplasm). Additionally, synthetic RNA can be mistakenly recognized as viral RNA by endosomal and intracellular receptors of the innate immune system (e.g., the Toll-like receptors TLR-3, TLR-8, and TLR-9; PKR and RIG-I receptors), eliciting an inflammatory antiviral immune response. The off-target effects of RNA interference can be mitigated by chemical modification of RNA nucleotides (e.g., 2′-O-Me, 2′-O-methoxyethyl, 2′-F, phosphorothioate, etc.). Although the entirely non-modified or lightly modified siRNAs can mediate *in vivo *gene suppression, extensive modifications can enhance the chemical stability and siRNA delivery efficiency, reduce the toxicity related to off-target effects, and decrease activation of the innate immune system [37, 38]. The off-target effects can also be minimized by careful selection of siRNA nucleotide sequences using* in silico *algorithms and software for siRNA design [39, 40].

**Delivery systems for interfering RNAs**

siRNA delivery systems that prevent RNA degradation by endogenous nucleases and ensure penetration through the biological barriers, as well as allow regulation of the rate of endosomal escape of siRNA, have been actively developed over the past two decades. Endosomal escape is a critically important step for siRNA activity, limiting both the rate and efficacy of RNA interference, since prolonged residence in endosomes causes RNA degradation [40, 41].

siRNAs can be delivered using lipid, inorganic (Si, Au, Ca3(PO4)2, and FexOy) and polymeric nanoparticles (chitosan, cyclodextrin, polyethyleneimine, and poly-Llysine), dendrimers (polypropyleneimine and polyamidoamine), carbon nanostructures (carbon nanotubes, quantum dots, and nanodiamonds), as well as peptide carriers and conjugates (antibodies, peptides, NAcGal, and cholesterol) [42, 43, 44].

Lipid nanoparticles are structures consisting predominantly of phospholipids. Nanoparticles can be either artificially engineered (liposomes) or obtained from bodily fluids (extracellular vesicles, EVs). These systems for delivering drugs into cells are biocompatible, biodegradable, and have been well-studied [45]. Extracellular vesicles can also be artificially engineered via chemical treatment of cells with actindestabilizing compounds (cytochalasins, latrunculins, etc.) or other agents causing irreversible, chemically induced plasma membrane blebbing (paraformaldehyde, N-ethylmaleimide, etc.) [46, 47].

Lipid nanoparticles having surface modifications that enhance their stability or targeting specificity (e.g., the commercially available ionized amphiphilic lipid nanoparticles for siRNA delivery DLin-DMA, DLin-MC3-DMA, and L319) are of the greatest interest [48]. The nanoparticle surface can be functionalized using various ligands: apolipoproteins, transferrins, folates, integrins, etc. PEGylation of the surface of siRNA-loaded liposomes was shown to ensure prolonged systemic circulation of lipid particles [33]. Additional functionalization of the nanoparticle surface with a peptide aptamer specific to fibronectin, whose expression on glioma cells is significantly upregulated, ensures targeted delivery of liposomes into tumor cells [49], tumor growth inhibition, and better survival of tumor-bearing animals. In another study, liposomal particles were functionalized with a ligand targeting LRP-1 (low-density lipoprotein receptorrelated protein 1). LRP-1 is expressed by blood– brain barrier endothelial cells and glioblastoma cells. It was demonstrated that these siRNA-MDK-loaded nanoparticles reduce the resistance of cancer cells to TMZ and inhibit tumor growth in orthotopic glioblastoma mouse models [50]. In the functionalization of lipid particles, ligands specific to αvβ3 and αvβ5 integrins were used to deliver siRNAs into tumor cells; αvβ6-specific ligands were utilized for siRNA delivery into lung epithelial cells in COVID-19 [51, 52].

siRNAs can be efficiently delivered only provided that the biological barriers impeding the penetration of positively charged particles are overcome [53]. The strategies to overcome the so-called “polycation dilemma” primarily involve designing surface charge-reversible nanoparticles. These ionizable lipid nanoparticles carry a moderately negative or neutral surface charge, which enhances their stability in bodily fluids. However, a shift in pH or the redox potential, or the action of endogenous enzymes and exogenous factors, leads these nanoparticles to change their surface charge to a positive one and be efficiently internalized by target cells [50, 53]. Hence, in order to be able to cross the BBB, liposomes can be shielded with catechol– polyethylene glycol polymers preventing the premature release of the liposomal cargo into the cytoplasm of non-target cells (endothelial cells, pericytes, etc.) [50]. The shielding is removed in a tumor characterized by an elevated level of reactive oxygen species, and these nanoparticles penetrate glioblastoma cells through the action of the targeting ligand.

Hybrid structures composed of liposomes and extracellular vesicles (EVs) have been proposed as an alternative approach to enhancing the targeting specificity of siRNA-loaded lipid nanoparticles. Extracellular vesicles are natural RNA carriers that are superior to liposomes due to their low toxicity and immunogenicity [54]. Extracellular vesicle surface markers can be displayed on the surface of these hybrid structures, making nanoparticles “inherit” their properties. For example, cardiac progenitor cells (CPCs) produce a variety of regulatory growth factors and cytokines. Hence, CPC-derived EVs activate endothelial cell migration and angiogenesis *in vivo*, which can be further utilized in developing cellular technologies to treat post-infarction conditions. Hybrid liposomal particles produced using CPC-derived EVs are also capable of activating endothelial cell migration [55]. The surface of EVs can be modified with molecules targeting them to specific cells, or EVs can be loaded with biologically active molecules (chemotherapeutics, growth factors, microRNA, or siRNA) [56]. Thus, the therapeutic effect of EV-siBRAFV600E was demonstrated in mouse models of colorectal cancer carrying the *BRAF *V600E mutation [57]. When producing, isolating, and characterizing extracellular vesicles, in order to increase the reproducibility and minimize side effects, one must strictly adhere to the “Minimal Information for Studies of Extracellular Vesicles” guidelines developed by the International Society for Extracellular Vesicles [58].

Hence, there is an ongoing effort focusing on the development of lipid systems for optimal intracellular drug delivery. The regulatory approval of patisiran (ONPATTRO, manufactured by Alnylam Pharmaceuticals), which is the PEGylated liposomal nanoparticle loaded with siRNA targeting coagulation factor VII (proconvertin), is one of the successful outcomes of this research [41].

The display of tissue- or organ-specific molecules on the surface of siRNA-loaded nanoparticles is also used in the case of non-lipid nanoparticles. Thus, it has been demonstrated that calcium phosphate nanoparticles “decorated” with apolipoprotein E3 can cross the blood–brain barrier and ensure efficient siRNA delivery, inhibiting the growth of tumor xenografts [59]. siRNA conjugates with N-acetylgalactosamine (NAcGal), a ligand binding to the asialoglycoprotein receptor specifically expressed on the hepatocyte surface, deserve special mention among polymeric nanoparticles. The interaction between these nanoparticles and hepatocytes induces rapid endocytosis and reduces the target mRNA levels in hepatocytes [60, 61]. Five out of the six siRNAbased therapeutics approved for clinical application (*Table 2*) are siRNA–NAcGal conjugates. However, they are less stable than liposomes and more difficult to manufacture [62, 63]. Compounds such as cholesterol [64], 2’-O-hexadecyl (C16) [65], aptamers [66], antibodies [67], and peptides [68] can be used as siRNA conjugates along with NAcGal.

Delivery using cell-penetrating peptides (CPPs) is a rapidly developing technology for siRNA delivery into cells. CPPs are usually short positively charged peptides capable of entering cells either via endocytosis or by directly crossing the membranes. CPPs were shown to be able to form non-covalent complexes or covalent conjugates with biologically active nucleic acids (including siRNAs) and ensure transfection of various cells [69, 70]. For example, a fragment of human kappa-casein, RL2, is capable of delivering plasmid DNA, small nucleolar RNA, and siRNA into cells. The most effective transfection was achieved by using the RL2–siRNA complexes; effective suppression of the expression of the target *EGFP *gene was demonstrated in ref. [71]. Despite all their advantages, CPPs also share the shortcomings inherent to proteinbased drugs, such as the short half-life, the challenges related to the optimization of the conditions for forming a monodisperse suspension of these particles, and high cost of production. Therefore, CPPs are used as components of hybrid particles (e.g., with PEG) or as antigens displayed on the surface of siRNA-loaded lipid nanoparticles [72, 73].

Hence, first on the list when developing siRNAbased therapeutics is to enhance the stability of the molecule in the internal environment of an organism. This can be achieved both via modification of the siRNA structure and through conjugation of siRNA with other compounds. Further optimization can involve encapsulation of siRNA into nanocarriers such as cationic liposomes or carbon nanostructures and incorporation of a targeting ligand. All these factors protect siRNA against the aggressive biological environment, increase the nanoparticles’ tropism towards the target, and, therefore, the effectiveness of RNA interference for a specific target gene.

**RNA interference as a promising approach to glioblastoma therapy**

**Fig. 1:**
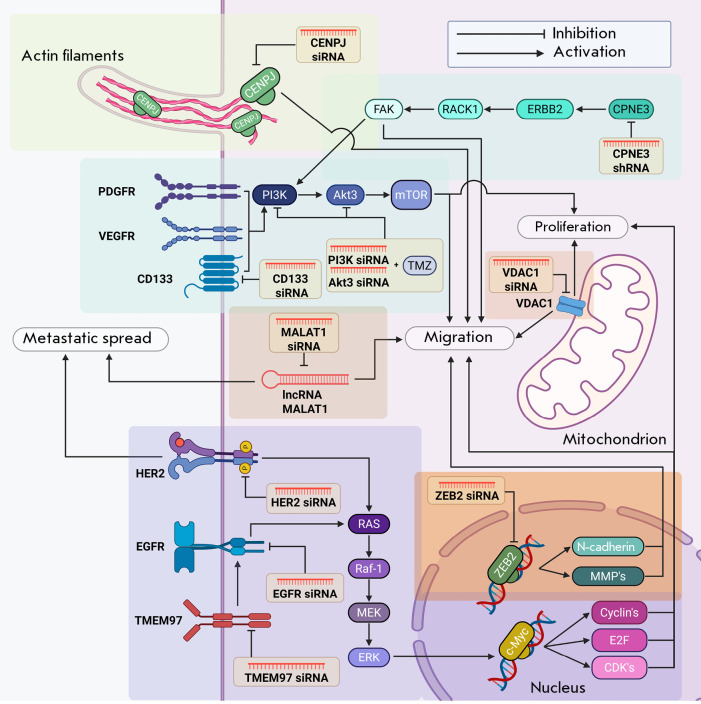
The use of RNA interference to regulate the expression of genes whose products are involved in the proliferation and migration of glioblastoma cells. siRNA – small interfering RNA; shRNA – short hairpin RNA

*In vitro *and *in vivo *experiments have demonstrated that the RNA interference technology is effective in inhibiting the signaling pathways that facilitate invasion, angiogenesis, and proliferation of glioblastoma cells, as well as their resistance to chemotherapy and radiotherapy. Thus, treatment of human glioblastoma T98G cells with siRNAs targeting the *Akt3 *and* PI3K *genes, in combination with temozolomide (TMZ), caused S and G2/M cell cycle arrest, in addition to inducing apoptosis and necrosis in tumor cells [74]. The PI3K/Akt/mTOR signaling pathway regulates apoptosis, proliferation, invasion, metabolism, epithelial–mesenchymal transition, and DNA repair in glioblastoma cells (*Fig. 1*) [75]. The PI3K/Akt/mTOR pathway is activated upon interaction of the epidermal growth factor (EGF), platelet-derived growth factor (PDGF), and vascular endothelial growth factor (VEGF) with their tyrosine kinase receptors. This signaling pathway was shown to be associated with the development of drug resistance, and its inhibition via RNA interference increased the sensitivity of U251 MG human glioblastoma cells to bortezomib [76].

The CD133 protein is considered to be a marker of cancer stem cells (CSCs), including glioblastoma stem cells [77]. The involvement of CD133 in oncogenesis makes it a crucial therapeutic target for the elimination of CSCs, which largely contribute to tumor recurrence, as well as for the inhibition of invasion, migration, and epithelial–mesenchymal transition. The activity of CD133–siRNA was shown to reduce the migration rate of U87 MG cells. This can be related to the modulation of the PI3K/Akt/mTOR signaling pathway (*Fig. 1*). In particular, RNA interference of the *CD133 *gene downregulated expression of the* RAF1*, *MAP2K1*, *MAPK3*, *PIK3CA*, *AKT3*, and *mTOR* genes [78].

Suppression of the expression of epidermal growth factor receptor (EGFR) and human epidermal growth factor receptor 2 (HER2) is another example of signaling pathway inhibition. These receptors mediate the activation of the MAPK/ERK signaling pathway, which regulates the proliferation and migration of cancer cells (*Fig. 1*). Thus, ERK activates transcription factors such as c-Myc, which in turn upregulate the expression of cell cycle regulator genes. The target genes of c-Myc include cyclin-dependent kinases, cyclins, and the transcription factor E2F [79]. HER2–siRNA was shown to reduce the migration and proliferation rates of LN-229 and U251 MG cells by approximately 50% [80]. Knockdown of the *EGFR* gene decreased the proliferation rate of both cell lines by approximately 40%. Expression of the *IGFBP3* gene, which belongs to the aforementioned MAG group (*Table 1*), is also modulated by the MAPK/ERK signaling pathway and positively correlates with cancer grade [24, 81]. The *in vivo *experiments on an orthotopic mouse model of U87 MG/Luc glioma showed that two siRNAs (siIBP3-1 and siIBP3-2) inhibited tumor growth. The *STAT3*, cofilin-1, galectin-1, and *ELTD1 *genes, which are also activated by the MAPK/ERK signaling pathway, are considered promising siRNA targets [82, 83].

A promising target for tumor therapy is the* TMEM97 *gene, which encodes the transmembrane protein TMEM97 (sigma-2 receptor (σ2R)) [84] and interacts with the EGF tyrosine kinase receptor (*Fig. 1*). Suppression of *TMEM97 *expression via RNA interference in U87 MG and U373 MG cells reduced the proliferation, migration, and invasion of cells, in addition to inducing G1/S cell cycle arrest [85]. Furthermore, RNA interference of the *TMEM97 *gene led to the modulation of epithelial–mesenchymal transition: the β-catenin and Twist levels declined, while the E-cadherin level increased.

Voltage-dependent anion-selective channel 1 (VDAC1) is a protein involved in non-selective transport of anions and cations across the outer mitochondrial membrane, as well as in the export of ATP into the cytoplasm (*Fig. 1*). The upregulated expression of the *VDAC1 *gene is known to play a crucial role in the reprogramming of metabolic and energy processes in cancer cells [86]. Inhibition of *VDAC1 *expression was shown to reduce the migration and invasion rates of human glioblastoma U87 MG cells *in vitro*, as well as slow down the growth of the U87 MG tumor in a mouse model [87, 88]. This is attributed to the dissipation of the mitochondrial membrane potential in tumor cells, reducing the intracellular ATP concentration and causing disruption of the cellular metabolism.

Along with protein-coding genes, the targets for gene-targeted therapy based on RNA interference can also include long non-coding RNAs (e.g., MALAT1 (*Fig. 1*), whose high expression level is associated with a poor prognosis in glioblastoma patients [89]). The MALAT1 levels were shown to be elevated in TMZ-resistant U251 MG and U87 MG human glioblastoma cells [90]. The cells, transfected with MALAT1–siRNA, were characterized by downregulated expression of the genes mediating drug resistance (*MDR1*, *MRP5*, and *LRP1*), as well as a downregulated expression of the *ZEB1 *gene, which is involved in the EMT in cancer cells. Tumor progression is accompanied by EMT associated with the degradation of the extracellular matrix and reduction of cancer cell adhesion, thereby intensifying their migration and invasion. Hence, the inhibition of these cellular processes via RNA interference can significantly reduce the metastatic potential of the tumor. Copine 3 (CPNE3), belonging to the CPNE family of Ca^2+^-dependent phospholipid-binding proteins, plays a crucial role in the EMT of human glioblastoma cells (*Fig. 1*). CPNE3 induces the EMT by activating the FAK signaling pathway, thus promoting invasion and migration of tumor cells. Suppression of *CPNE3 *expression using CPNE3–shRNA in U87 MG and U251 MG cells impaired the migratory, invasive, and proliferative potential of glioblastoma cells, which can be associated with inactivation of the FAK and, therefore, the PI3K/Akt/mTOR signaling pathways [91, 92].

The ZEB2 protein is a transcription factor playing an important role in the development of the central nervous system throughout the entire embryonic period. Meanwhile, ZEB2 is also involved in the epithelial– mesenchymal transition of tumor cells; upregulated* ZEB2 *expression is observed in many cancers, including glioblastoma [63]. An analysis of the migratory potential of U87 MG and U373 MG glioma cells revealed that the migration rate of cells transfected with ZEB2–siRNA was significantly reduced compared to the control cells [93]. *ZEB2 *overexpression is known to increase the levels of N-cadherin and a number of matrix metalloproteinases (*Fig. 1*); in turn, it promotes invasion/migration of cancer cells [93, 94, 95]. The centromere protein J (CENPJ) controlling the division of neural precursor cells and neuronal migration is also involved in the EMT [96]. *CENPJ *expression was shown to be upregulated in human glioblastoma cell lines compared to healthy brain tissue; this correlates with a poor disease prognosis in glioma patients. Treatment of personalized glioblastoma culture cells (GBM02 and GBM95) with CENPJ–siRNA reduced their migration rate. *CENPJ *knockdown is believed to alter the morphology of glioblastoma cells because of microtubule stabilization and actin microfilament depolymerization, thereby making the cells less prone to epithelial–mesenchymal transition (*Fig. 1*).

NU-0129, a siRNA-based therapeutic designed for glioblastoma treatment, is currently undergoing phase I clinical trials (Clinical trials: NCT03020017). The therapeutic is a complex of gold nanoparticles and siRNA targeting *Bcl2L12 *mRNA. The *Bcl2L12* gene encodes the anti-apoptotic protein Bcl2L12 overexpressed in human glioma cells, which makes them apoptosis-resistant. An analysis of the accumulation of gold particles in patients’ tumors demonstrated that NU-0129 penetrates the blood–brain barrier and accumulates in tumor tissue, where it reduces the level of the Bcl2L12 protein [97]. Hence, designing targeted nontoxic nanoparticles carrying siRNAs described above and further research into their effectiveness for glioblastoma treatment is undoubtedly promising; the clinical trials of the developed drugs will broaden the treatment options for neuro-oncological disorders.

## CONCLUSIONS

RNA interference is a promising therapeutic approach to glioblastoma treatment. The currently available promising delivery systems for interfering RNAs lay the groundwork for designing targeted agents that inhibit the proliferation, invasion, migration, and epithelial–mesenchymal transition of tumor cells. The previously described signatures of the MAG genes, as well as the genes encoding the FAK, PI3K/Akt/mTOR, and MAPK/ERK signaling pathways, will facilitate the search for siRNAs with a potential for developing effective targeted therapies for glioblastoma. When developing siRNA-based therapeutics, our efforts should focus on enhancing their penetration efficiency, stability, and specificity with respect to a selected target.

## Abbreviations

BBB: blood-brain barrier
EMT: epithelial-mesenchymal transition
CD133: prominin-1
CENPJ: centromere protein J
CPC: cardiac progenitor cells
CPNE3: copine 3
EGF: epidermal growth factor
EGFR: epidermal growth factor receptor
EMA: European Medicines Agency
EV: extracellular vesicle
FAK: focal adhesion kinase
FDA: U.S. Food and Drug Administration
HER2: human epidermal growth factor receptor 2
IDH: isocitrate dehydrogenase
MAGs: metastasis-associated genes
MALAT1: metastasis-associated lung adenocarcinoma transcript 1
mCSCs: metastatic cancer stem cells
MDK: midkine (cell growth factor)
MMPs: matrix metalloproteinases
NAcGal: N-acetylgalactosamine
PDGF: platelet-derived growth factor
RISC: RNA-induced silencing complex
siRNA: small interfering RNA
shRNA: short hairpin RNA
TMZ: temozolomide
VDAC1: voltage-dependent anion channel 1
VEGF: vascular endothelial growth factor
